# Diet composition and preferences of Bohor reedbuck (*Redunca redunca* ) in the compound of Alage College, Central Rift Valley of Ethiopia

**DOI:** 10.1002/ece3.6939

**Published:** 2020-11-03

**Authors:** Yonas Derebe, Zerihun Girma

**Affiliations:** ^1^ Department of Forest and Climate Science Injibara University Injibara Ethiopia; ^2^ Department of Wildlife and Protected Area Management Hawassa University Hawassa Ethiopia

**Keywords:** feeding preference, focal animal, grazing, laboratory analysis

## Abstract

Numerous indices have been developed to compare use and availability of foods in field diets of wild ungulates. However, little attention has been given to laboratory analysis for comparing food preferences. To this end, a study aimed at investigating the diet composition and preference of Bohor reedbuck was conducted in the compound of Alage Agricultural College, Central Rift Valley of Ethiopia from 2017 to 2018 encompassing both dry and wet seasons. Bohor reedbuck is a medium sized horned antelope species endemic to Africa. Continuous focal animal observation was used to collect the data on plant species included in the diet of Bohor reedbuck. Focal individuals’ observation was carried out for 30 min in 10 min sampling interval during their active feeding period (early morning and late afternoon) over four different habitat types. The nutrient composition of plants consumed was determined using wet chemistry laboratory analysis. Bohor reedbucks consumed 15 species of plants; herbs comprised 94.3% of the foods they consumed. Digitaria abyssinica was the most preferred plant species with highest crude protein (23.75%) and less fiber (61.8% nitrogen detergent fiber and 27.8% acid detergent fiber). These findings suggest that food preference of Bohor reedbuck is determined by the nutritional content of the plant it consumed, since the area is more or less natural habitat in terms of plant species composition. For sustainable conservation of the species, there is a need to actively promote management of the plant species most preferred by the reedbuck to feed on.

## Graphical abstract

Little attention has been given to laboratory/proximate analysis/approaches for comparing food preferences of Ungulates, particularly Bohor reedbucks. Bohor reedbucks preferred to consume plants with high crude protein content and less fiber. Sustainable conservation of Bohor reedbuck demands the effective management of selectively foraged plants.

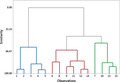

## 1 INTRODUCTION

Bohor reedbuck (*Redunca redunca*) is a medium‐sized African endemic antelope species placed under the genus *Redunca*, family Bovidae (Huffman, 2013; IUCN SSC Antelope specialist group, 2008). It was first described by German zoologist and botanist Peter Simon Pallas in 1767 (Estes, 1991; IUCN SSC Antelope Specialist Group, 2008). Five sub‐species of Bohor reedbuck are known namely *R*. *redunca redunca*, *R*. *r*. *nigeriensis*, *R*. *r*. *cottoni*, *R*. *r*. *bohor,* and *R*. *r*. *wardi*. The five sub‐species of Bohor reedbuck are mostly known to occur in equatorial region of Africa (from Senegal (west) to Ethiopia (east)) (Kingdon, 2015). Among the five sub‐species of Bohor reedbuck, *Redunca redunca bohor* is found in west, central, and southeast Ethiopia and Blue Nile of Sudan.

In Ethiopia, Bohor reedbuck populations have wide distribution from lowlands to high peak mountains (Afework et al., 2009; Girma, 2018; Habtamu et al., 2012; Yalden et al., 1984). They have been recorded as low as 400 m asl in the woodland and floodplain grasslands and as high as above 3,200 m asl in the open, swamp, and bushy montane grasslands (Afework et al., 2009; East, 1999; Yalden et al., 1984). Bohor Reedbuck prefers water abundant areas and associated inundated grasslands (Kingdon & Hoffmann, 2013). Bohor reedbucks are mixed feeders. Open grassland and swamp grasslands are the preferred feeding sites of Bohor reedbucks. They are herbivore, they prefer grasses and tender reed shoots with high‐protein and low‐fiber content (Estes, 1991). The feeding ecology of Bohor reedbuck varies in season, for example in the dry season the diet of Bohor reedbuck consisted mainly of dicots, and they eat forbs and leaves of woody plants (Estes, 1991; Gutbrodt, 2006), and also, the amount of time spent while grazing in a particular area is possibly related to the availability and quality of grasses there in different season (Kingdon & Hoffmann, 2013).

Numerous food preference indices have been developed to compare use and availability of foods in the field of study of diets of wild ungulates. However, little attention has been given to laboratory analysis for comparing food preferences of a species across different ecosystems, regions, among others (Girma, 2016; Rodgers, 1990). Little is known about the nutritional content of plants consumed by Bohor reedbucks and how food nutritional composition determines their diet selection. Studies on feeding ecology of Bohor reedbuck in Ethiopia addressed only the diet composition and preferences without addressing the nutritional composition of the plants consumed by the species (Afework et al., 2009; Habtamu et al., 2012).

Feeding ecology is vital ecological phenomenon that describes the interaction between plant communities and herbivores. Foraging behavior is governed by various plant attributes such as plant availability and palatability and plant nutritional composition (Clauss et al., 2010; Tanentzap et al., 2009). Studies have indicated that selective foraging on high‐protein and low‐fiber content plants boost nutrient and energy intake and also decrease retention time, ultimately increasing intake capacity and fitness (Mysterud et al., 2001; Zweifel‐Schielly et al., 2012). It has been reported that for herbivores the level of nutrients and toxins in a plant or its neighbors are key factors that determine the palatability of the plant to be consumed (Provenza et al., 2002). Since, all plants contain some amount of toxin herbivores cannot completely avoid toxins, but have to regulate the intake of toxins (Foley et al., 1999). Ultimately the interaction between plant chemistry and herbivore learning determines the coexistence of plants and animals affecting sustainable biodiversity conservation (Provenza, Villalba, Dziba, Atwood, & Banner, 2003). Furthermore, animal factors such as body size, digestive physiology and experience determine foraging behavior (Clauss & Hummel, 2005; Clauss et al., 2008; Parker & Bernard, 2006).

Studies on feeding behavior are necessary to enable estimates of the coexistence of wild animals in a particular ecosystem (Gutbrodt, 2006). The behavior of individuals and the strategies they adopt to maximize fitness plays an important role when a species’ natural behavior can lead to conservation problems in habitats altered by humans (Festa‐Bianchet & Marco, 2003). Information on nutritional composition of herbivores is vital for better understanding of resource requirements and offers intuition into herbivore influences on an ecosystem as well as animal populations (Parker & Bernard, 2006; Tanentzap et al., 2009). Clear nutritional composition analysis is crucial for clear understanding of animal normal growth and the maintenance of good health (Clauss et al., 2010). Therefore, this study aimed to determine the nutritional composition and preferences of Bohor reedbuck in the compound of Alage Agricultural College (AAC), Central Rift Valley of Ethiopia.

## 2 MATERIALS AND METHODS

### 2.1 Description of the study area

**FIGURE 1:**
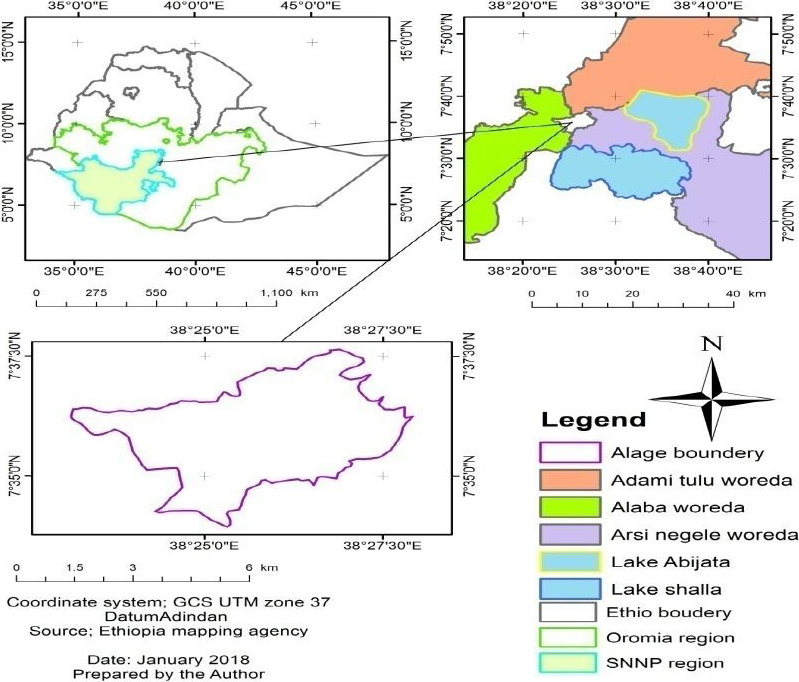
Location map of Alage Agricultural College

Alage Agricultural College is located at about 217 km south west of Addis Ababa in Central Rift Valley zone of Ethiopia. The college shares its boundaries between two administrative regions, namely, Oromia and South Nations Nationalities and Peoples Regional States (Figure 1). The study area is geographically situated between 7°35′0′′ to 7°37′30′′N latitude and 38°25′0′′ to 38°27′30′′E longitude (Figure 1).

The topography of the area is characterized by midland with altitude ranging from 1,580 to 1,650 m above sea level. The area is characterized by bimodal rainfall pattern. It receives most of its rain in the months of June to September, with minor erratic rainy season during the months of March and April. The average annual rainfall is 708–900 mm (Ethiopian National Meteorological Agency, 2017). The mean maximum and minimum annual temperature is 30.19°C and 8.1°C respectively (Ethiopian National Meteorological Agency, 2017).

The total area of the college is 29.46 km^2^. Jido River surrounds the vast majority of the area in the north, east and north east directions. As described by Water Works Design and Supervision Enterprise (WWDSE) (WWDSE, 2013) the land cover is dominated by acacia wooded grassland (55%) followed by riverine and plantation forest (6.1%), open grasslands (8.4%) and farmlands, residential and other service areas (19.2%). Only the small part of the college is fenced. Furthermore, there was no management plan for flora and fauna. Particularly, there were no stocking plan for vertebrates and planting plan for vegetation (WWDSE, 2013), implying that the study area is more or less natural habitat.

Dominant tree species in the area are; *Acacia seyal*, *Dicrostaches cineria*, *Acacia tortolis*, *Acacia melifera*, *Acacia macrostachia*, *Eucaluptus spp*, *Balanite aegyptica,* and *Aloe spp*. In addition, characteristic grass species such as *Cynodon aethiopicus*, *Hyparrahenia cymbaria*, *Cynodon dactylon*, *Chloris gayana,* and *Panicum maximum* dominate the open grassland areas (WWDSE, 2013). Mammals such as warthog (*Phacochoerus aethiopicus*), Bohor reedbuck (*Redunca redunca bohor*), African civet cat (*Civeticus civetta*), spotted hyena (*Crocuta crocuta*), grivet monkey (*Cercopithecus aethiops*), Abyssinian hare (*Lepus habessinicus*), and anubis baboon (*Papio anubis*) are frequently sighted from the area (First author personal observation, 2018).

### 2.2 Methods

#### 2.2.1 Sampling design

Four easily recognizable and approachable groups of Bohor reedbuck populations were selected during reconnaissance survey representing the four dominant habitat types namely; Acacia dominated wooded grassland, Riverine forest, Open grass land and Farm land. Recognizable groups (known group size, natural marks, known home range and activity) of Bohor reedbuck were selected and observed in each of the four different habitat types. Individuals observed up to 50 m far from the group was counted as the member of the same group (Caro, 1999).

The dietary data of Bohor reedbuck were collected through continuous focal animals observation of individuals (ranged from 5 to seven individuals per group, the number of individuals varied based on the habitat type) of the same group that were not reproductively active (all individuals targeted were available during the whole data collection period) (Altmann, 1974). Focal animals’ observation was carried out through following up all individuals of one selected easily recognizable and approachable groups of Bohor reedbuck population at a time (Yihune & Bekele, 2012). Observation of focal individuals was carried out for 30 min in 10 min sampling interval during morning hours (7:00 a.m. to 11:00 a.m.) and late afternoon hours (3:00 p.m. to 5:00 p.m.) when the animals were actively foraging, for each recognizable group at each habitat type during both dry and wet seasons. A total of 82 hr in 10 days (both in wet and dry seasons) were spent observing the focal animals. In each season a total of 41 hr in five days were spent observing the focal animals. For each group in each habitat type equal numbers of hours were spent observing the focal animals. The observations were carried out with a distance (between 50 and 200 m) approaching the group from different directions (Afework et al., 2009; Tekalign & Bekele, 2012). All individuals in a group were observed at the same time with a help of trained personnel of the Bohor reedbuck identification techniques, the method of sampling and plant consumed. Field assistants were B.Sc. graduates in wildlife management and related natural resource management disciplines.

The frequency of occurrence of the plant species in the study area that was consumed by Bohor reedbuck and not consumed but available to Bohor reedbuck was estimated using 21 plots (4 m by 4 m) established along 7 transect lines. Transect lines and sampling plots were established proportionally to the size of each habitat type. Accordingly in Acacia dominated wooded grass land 3 transects and 12 plots were established, whereas in riverine, open grasslands and farmlands 1 line transect that consist of 3 sampling plots in each habitat type were established. Transects and plots were established representing each habitat types and in a way it captures all of the plants consumed by the Bohor reedbuck. The area is sparse in its vegetation diversity (mostly covered by bare soil), and few sampling plots could exhaustively capture the available plant species in the area. The distance between each transect were 0.5 km, and also, the distance between each plot was 0. 5 km and the length of each transect was 2 km.

#### 2.2.2 Data collection

The data on groups of feeding Bohor reedbuck were recorded particularly; the type of plant species consumed and the type of food item preferred (young leaves, mature leaves, shoots, flowers and fruits) during both wet and dry seasons. To minimize any disturbance effect caused by the observer camouflaging clothes were used. Furthermore, the animals were approached walking gently and quietly against the direction of wind movement to avoid being scented by the animals. Once the animals were approached to observable distance, before collecting information they were allowed to become acclimated and resume feeding for a minimum of 10 min following Hochman and Kotler (2006), Taddesse and Kotler (2011), Taddesse and Kotler (2013), & Girma (2016). Close investigation of the feeding site were made for verification of plant species consumed. Close investigation of the feeding site was carried out after the 30 min continuous observation ended up during 10 min interval break. Evident, green, moist, freshly severed tissue characteristics were considered as a confirmation of a particular plant species consumed by the ungulates (Girma, 2016; Pienaar, 2013). The plant species were identified by the guide; Flora of Ethiopia and Eritrea (Phillips, 1995) and by comparison with archived specimens from the collection housed at the National Herbarium in Addis Ababa University.

The untouched fresh (both young and mature leaves, but not old dried ones) plant leaves and shoots, flowers and fruits for only grass species consumed by the Bohor reedbuck from the four habitat types were collected in a plastic bag for the proximate analysis. All plant parts were mixed together for the proximate analysis, no classifications in to plant parts were made. Forage quality analysis was carried out based on wet season data and across habitat due to logistic constraints. Thus, seasonal or habitat variations were not tested. The proximate analysis of 15 plant species (five from highly preferred, five from moderately preferred and 5 from rarely consumed and avoided) consumed by Bohor reedbuck was used as forage quality test. As part of the proximate analysis Moisture, Dry Matter, Organic Dry Matter, Ash, Nitrogen/Crude protein, Neutral Detergent Fiber (NDF), and Acid Detergent Fiber (ADF) was analyzed following AOAC (1995) analytical procedures. According to AOAC (1995) the following procedures were used to carry out the laboratory analysis for all proximate analysis.

Before proceeding to the analysis the weight of sampled items were weighed using electronic balance and then fresh forage samples were dried for 48 hr at 60°C in an air‐circulation oven to obtain air dried samples ready for grinding. The weight of dried sample was recorded with electronic balance and also the dried samples were grounded to 1mm particle size with a Wiley mill. Dried and ground samples were stored in airtight containers away from heat and light to avoid moisture in take. The dry matter content of the sample plant were determined through adding approximately 2 g of ground sample to the crucible and drying it overnight at 105°C with in the oven dry, then the percentage of dry matter (DM) was calculated. The ash content of the sample was determined by igniting dry matter samples overnight at 550°C in muffle furnace, and then percent ash was calculated. Determination of total nitrogen (crude protein) was conducted mixing 0.2g sample with equal amount of catalyst and digesting the sample in sulfuric acid using K_2_SO_4_/ CuSO_4_ as a catalyst. Then, N was converted into NH_3_, then the distilled solution was trapped in boric acid and titrated with H_2_SO_4_, then Percent of Crude Protein (CP) was calculated. Determination of the Neutral Detergent Fiber (NDF) was carried out through adding 0.5 g sample (Ws) in 600 ml Berzelius beaker and adding 100 ml of neutral detergent solution and 0.5 g sodium sulfite (Na_2_SO_3_). Then the mixture was boiled for one hour in refluxing apparatus and was poured through glass crucibles and vacuum was admitted, residues were ashed for three hours at 550°C and cooled to room temperature in desiccators. Determination of Acid Detergent Fiber (ADF) was conducted through adding 0.5 g sample (Ws) and 100 ml of acid detergent in 600 ml Berzelius beaker solution. Then the mixture was boiled for one hour in refluxing apparatus and poured through glass crucibles and admitted vacuum, ash residues for three hours at 550°C and cool to room temperature in desiccators.

The relative density and frequency of occurrence of the plant species that was consumed by the Bohor reedbuck and not consumed, but available to Bohor reedbuck were recorded through estimating their density and occurrence in different plots and extrapolating the average to the size of the total study area.

#### 2.2.3 Data analysis

Microsoft Excel 2010 was used for data summarization and to organize results into tables and figures. All statistical testes were analyzed using SPSS version 23 computer software program. Mann–Whitney rank test analysis was used to examine the relationship between relative frequency of consumption of species and its availability (i.e., relative frequency of occurrence) following Johnson (1980).

The food preference indices (FPI) for each plant species consumed by Bohor reedbuck was calculated and ranked to determine the most preferred plants. The feeding preference index (FPI) of Bohor reedbuck for each plant species was calculated by the Equations ((1), (2), (3));

(1) $$\mathrm{FPI}=\frac{\mathrm{frequency}\mathrm{of}\mathrm{usage}\mathrm{the}\mathrm{plant}\mathrm{species}}{\mathrm{frequency}\mathrm{of}\mathrm{occurrence}}$$

(2) $$\text{Frequency}\;\text{of}\;\text{usage}\;\text{of}\;\text{the}\;\text{plant}\;\text{species}=\frac{\text{number}\;\text{of}\;\text{record}\;\text{the}\;\text{plant}\;\text{species}\;\text{consumed}}{\text{Total}\;\text{number}\;\text{of}\;\text{sample}\;\text{records}\;}$$

The frequency of occurrence (FO) of the plant species in the area also was calculated using equation (3) below

(3) $$\mathrm{FO}=\frac{\mathrm{Number}\mathrm{of}\mathrm{plots}\mathrm{the}\mathrm{species}\mathrm{ocurr}}{\mathrm{Total}\mathrm{number}\mathrm{of}\mathrm{plots}}$$

The absolute density (density) of the plant species in the area is calculated using Equation (4) below

(4) $$\mathrm{Density}=\frac{\mathrm{Number}\mathrm{of}\mathrm{individuals}}{\mathrm{Sum}\mathrm{of}\mathrm{plot}\mathrm{areas}}$$

The Relative Density (RD) of the plant species in the area was calculated by Equation (5);

(5) $$\text{RD}=\frac{\text{Total estimated density of a given specie*}100}{\text{Total density of all consumed species}}$$

Percentage preference of Bohor reedbuck to plant parts (%PPP) was computed by Equation (6);

(6) $$\%\text{PPP}=\frac{\text{Number of samples the plant parts consumed}\times 100}{\text{Number of samples the plant spp}.\;\text{consumed}}$$

Sorenson's similarity (Ss) index between the plant species consumed by Bohor reedbuck during wet and dry season was made following (Kent & Coker, 1992) (Equation 7).

(7) $$\text{Ss}=\frac{2a}{\left(2a+b+c\right)}$$

where *a* = number of species consumed in both seasons, *b* = number of species unique to wet season *c* = number of species unique to dry season.

Chi‐square test was used to examine frequency of each plant species consumed among habitat type.

According to AOAC (1995) the percentage values of all the proximate analysis were calculated by the Equations (8)–(14);

- $\%M=\left[\frac{{(w}_{\mathrm{bd}}‐w_{\mathrm{ad}})\times 100}{w_{s}}\right]$ (8) (Method 930.04)

where %*M* = percentage of Moisture, *W*_bd_ = wt of sample + dish before drying, *W*_ad_ = wt of sample + dish after drying, *W*s = Wt of sample taken.

- $\%\mathrm{DM}=\left[\frac{{(w}_{0}‐w_{t})\times 100}{w_{s}}\right]\mathrm{or}\%\mathrm{DM}=100‐\%\mathrm{Moisture}$ (9) (Method 2001.2)

where %DM = Percentage of dry matter, *W*_0_ = Weight oven–dry crucible + sample, *W*_t_ = Weight oven‐dry crucible, and *W*_s_ = record weight.

- $\%\mathrm{Ash}=\left[\frac{{(w}_{a}‐w_{t})\times 100}{w_{0}‐w_{t}}\right]$ (10) (Method 942.05)

where *W*_a_ = Weigh ignited crucible + sample, *W*_t_ = Weight oven‐dry crucible, *W*_0_ = (Weight oven–dry crucible + sample), *W*_t_ = Weight oven‐dry crucible

- $\text{percentage of Organic matter}\left(\%\;\text{OM}\right)=100‐\%\text{Ash}$ (11) (Method 967.05)
- $\%\;\text{CP}=\%N\times F,\;\%N=\left[\frac{1.4007\times \left(V_{a}‐V_{b}\right)\times N}{w}\right],\;F=6.25$ (12) (Method 978.04)

where percentage of Crude protein (%CP), %*N* (total nitrogen in sample), *F* (conversion factor); *F* = 6.25, *V*_a_: volume of acid used for sample titration, *V*_b_: volume of acid used for the blank, *N*: Normality of acid, *W*: sample weight in grams and 1.4007: conversion factor mill equivalent weight of nitrogen and N percent

- $\%\;\text{NDF}=\left[\frac{(w_{0}‐w_{t})\times 100}{w_{s}}\right]$ (13) (Method 2002.04)

where percentage of Neutral detergent fiber (%NDF), *W*_0_ = Weigh sample and crucible, *W*_t_ = Weigh oven‐dry glass crucible and *W*_s_ = weight of sample;

- $\%\text{ADF}=\left[\frac{(w_{0}‐w_{t})\times 100}{w_{s}}\right]$ (14) (Method 973.18)

where Percentage of Acid detergent fiber (ADF), *W*_0_ = Weigh sample and crucible, *W*_t_ = Weigh oven‐dry glass crucible and *W*_s_ = weight of sample.

Since nutrient requirement data for Bohor reedbuck is not available, the nutrient requirements of common bushbuck (Macleod et al., 1996) were used to determine an ideal or optimal nutritional profile. Common bushbuck was selected due to availability of published data on nutritional profile and relatively closes relation of the species to Bohor reedbuck.

Multivariate hierarchical clustering procedure was carried out to classify the plant species consumed by Bohor reedbuck into forage quality groups based on percent crude protein and moisture content from proximate analysis and percent frequency of usage of plants consumed data calculated using the formula above (Equation 2). The amalgamation steps used the Euclidean distance and complete linkage (furthest neighbor) to calculate the inter‐cluster distances and the final partitioning presented in a Dendrogram.

## 3 RESULTS AND DISCUSSION

### 3.1 Diet composition

Bohor reedbucks consumed a total of 15 species of plants out of 35 plant species available in the study area (WWDSE, 2013); 12 species of herbs, 1 shrub and 2 tree species. The selective feeding strategy of Bohor reedbuck (Gutbrodt, 2006) could be the reason for reduced (15) number of plant species in their feed. Similarly, greater kudu selected 17 plant species out of 38 species available in the miombo woodland adjacent to Umfurudzi Park in Zimbabwe (Chinomona et al., 2018). Furthermore, elephants have been observed to selectively browse on 22 plant species out of 35 plant species available (Biru & Bekele, 2012). However, disparate finding to this study is a study conducted in Dinder National Park of Sudan indicated that Bohor reedbucks observed to consume a wide variety (25) of plant species in their diets (more than 85% of available plants) (Ahmed, 2005). Similarly, the generalist feeder common warthog has been observed to forage on about 83% of available plants in Tanzanian savanna (Treydte et al., 2006). The reduced variety of plant consumed in the present area could be attributed to the reduced floral diversity and poor nutritional quality of plants available in the present study area (WWDSE, 2013). The present study area is characterized by infertile sandy soil and erratic rainfall that promote sparse vegetation diversity and less nutritional quality feed. Furthermore, since the area is less protected as compared to Dinder National Park disturbances such as human activities, livestock grazing could reduce both the diversity of plant species and foraging opportunity of the species. The majority (50%–55%) of plant species avoided by Bohor reedbuck in the study area was lowest in nutritional composition (low crude protein, which is likely accompanied by increased carbohydrate) as compared to the benchmarked species common bushbuck (Macleod et al., 1996) and rarely occurs during dry season (WWDSE, 2013). Furthermore, as the dry season progresses the nutritional content (protein content, which is likely accompanied by increased carbohydrate) of the forage species decline and hence, become less preferred and attractive (Omphile et al., 2004). Several studies have indicated that floral diversity of a particular area may influence feeding composition of ungulates and shape their selective feeding behavior (Clauss et al., 2007; Codron et al., 2007; Owen‐Smith, 1994). Bohor reedbucks were both grazers and browsers but they were more grazers than browsers. Grasses comprised 73% of the plant species consumed by Bohor reedbuck and it contributed 94.3% of dry matter of their feed (Table 1). The predominant selective grazing of grass species over other plant species available by Bohor reedbuck was an indicator of their grazing behavior (Cerling et al., 2003; Estes, 1991; Kingdon, 1997). Similarly, Ahmed (2005) and Gutbrodt (2006) reported grasses comprised the highest proportion of Bohor reedbuck feed. The selective grazing behavior of the species implies the need for conservation of the selectively grazed plant species in the compound of the college for sustainable conservation of populations of Bohor reedbuck in the college.

**TABLE 1:** Plant species consumed by Bohor reedbuck during wet and dry seasons at Alage Agricultural College, Ethiopia

Plant species	Family	Habit/growth form	Seasons	Relative density (%)
*Cynodon aethiopicus*	Poaceae	Herb	Both	21.27
*Cynodon dactylon*	Poaceae	Herb	Both	20.73
*Chloris gayana*	Poaceae	Herb	Both	12.04
*Panicum maximum*	Poaceae	Herb	Both	8.83
*Dactyoctenium aegyptium*	Poaceae	Herb	Both	6.17
*Panicum infestum*	Poaceae	Herb	Dry	5.38
*Bothriochloa insculpta*	Poaceae	Herb	Both	5.21
*Pennisetum ciliare*	Poaceae	Herb	Dry	4.45
*Cenchrus cilaries*	Poaceae	Herb	Wet	3.55
*Digitaria abyssinica*	Poaceae	Herb	Both	3.33
*Hyparrhinia hirta*	Poaceae	Herb	Wet	3.1
*Acacia seyal*	Fabaceae	Tree	Dry	2.22
*Rhynchosia minima*	Fabaceae	Herb	Both	1.38
*Rhus quartiniana*	Anacardiaceae	Shrub	Both	1.18
*Balanites aegyptiaca*	Balanitaceae	Tree	Dry	1.1

Note

“Both” indicate the plant species consumed during the two (wet and dry) seasons.

A total of 11 and 13 species of plants were consumed during the wet and dry seasons respectively (Table 1). Despite the fact that plant species *Panicum infestum*, *Pennisetum ciliare*, *Acacia seyal,* and *Balanites aegyptiaca* were available in the study area during both dry and wet seasons, they were only consumed during dry season (Table 1). *Cenchrus cilaries* and *Hyparrhinia hirta* were available as forage only during wet season and consequently consumed only during wet season (Table 1). Sorenson's similarity index of plant species consumed by Bohor reedbuck during wet and dry seasons was 0.75. According to Ratliff (1993), Sorenson's similarity index values in a range of 0.51 to 0.75 indicates high similarly of species, implying considerably high degree of Bohor reedbuck dietary overlap between wet and dry seasons in the study area. Similarly, about 60% dietary overlap was recorded in the diet of Bohor reedbuck between dry and wet seasons in humid coastal savanna in Tanzania (Halsdorf, 2011). The significantly high diet overlap between dry and wet seasons could be explained by the year round availability of most consumed species in the study area. The slight increase in plant species consumed during dry season could be due to the fact that during dry season, the forage availability and quality of most plant species consumed decline and, hence the species had to include few more species to meet up the compromised forage quality and availability. Since the area receives little rainfall during dry season (WWDSE, 2013), grass species like *Pennisetum ciliares* and *Hyparrhinia hirta* were dried and were not readily available for consumption. The number of plant species in the diet of Bohor reedbucks increased during the dry season coinciding with decreasing quality of available forage (Omphile et al., 2004; Wolfson & Tainton, 1999). As selective feeders Bohor reedbuck depend on sufficient food quality and select the nutrient richest forage (Gutbrodt, 2006), it may include alternative foods during the dry season to meet their nutritional requirements.

The findings of feeding preference and proximate analysis indicated that crude protein and moisture content were the potential driving forces for selective grazing/browsing (Tables 2 and 3). Bohor reedbucks were selecting for a consistent nutrient profile (mainly crude protein and moisture) each season. Species highly preferred were those with relatively higher crude protein and high moisture contents during both dry and wet seasons. However, during dry season moisture content was found to highly determine selective foraging. For instance, *Acacia seyal* was exclusively browsed in dry season due to its better moisture content potential, despite its lowest crude protein content. *Acacia seyal* is typical arid tree species that conserve moisture in its leaves due small sized and waxy cuticle (Mohammed & Röhle, 2011).

**TABLE 2:** Feeding preference indices (FPI) of plant species consumed by Bohor reedbuck based on frequency of usage and occurrence at Alage Agricultural College, Ethiopia

Plant species	Frequency of usage	Rank	Frequency of occurrence	Rank	FPI	Rank
*Digitaria abyssinica*	0.56	3	0.47	9	1.19	1
*Cynodon dactylon*	0.89	1	0.79	3	1.13	2
*Panicum maximum*	0.57	2	0.75	4	0.76	3
*Chloris gayana*	0.54	4	0.81	2	0.67	4
*Dactyoctenium aegyptium*	0.35	5	0.55	6	0.64	5
*Panicum infestum*	0.26	6	0.51	7	0.51	6
*Balanites aegyptiaca*	0.04	14	0.08	15	0.50	7
*Bothriochlo ainsculpta*	0.26	7	0.68	5	0.38	8
*Pennisetum ciliare*	0.15	9	0.45	10	0.33	9
*Rhus quartiniana*	0.05	13	0.16	14	0.31	10
*Cynodon aethiopicus*	0.24	8	0.90	1	0.27	11
*Hyparrhinia hirta*	0.09	11	0.39	11	0.23	12
*Rhynchosia minima*	0.06	12	0.26	12	0.23	12
*Cenchrus cilaries*	0.10	10	0.53	8	0.19	13
*Acacia seyal*	0.04	15	0.23	13	0.17	14

Note

Rank usage refers to the frequency of use of a particular plant species by the Bohor reedbuck, and rank FPI is a factor of frequency of use and availability of a particular plant species consumed by the Bohor reedbuck.

**TABLE 3:** Proximate analysis of plants species consumed by Bohor reedbuck at Alage Agricultural College, Ethiopia

Plant species	%M	%DM	%Ash	%ODM	%CP	%NDF	%ADF	FPI
*Digitaria abyssinica*	13.6	86.4	19.37	80.63	23.75	61.19	27.8	1
*Cynodon dactylon*	11.71	88.29	19.32	80.68	18.56	66.75	30.3	2
*Panicum maximum*	11.66	88.34	21.23	78.77	11.02	62.64	49.3	3
*Chloris gayana*	12.98	87.02	21.83	78.17	21.17	69.77	34.5	4
*Dactyoctenium aegyptium*	14.07	85.93	18.39	81.61	21.74	67.01	42.2	5
*Panicum infestum*	16.51	83.49	19.78	80.22	14.43	60.62	45.0	6
*Balanites aegyptiaca*	17.53	82.47	20.12	79.88	17.94	28.91	31.0	7
*Bothriochloa insculpta*	10.08	89.92	20.06	79.94	17.5	67.77	37.1	8
*Pennisetum ciliare*	16.97	83.03	19.99	80.01	9.89	52.99	39.9	9
*Rhus quartiniana*	11.83	88.17	14.01	85.99	21.85	33.17	34.7	10
*Cynodon aethiopicus*	13.11	86.89	15.64	84.36	10.41	47.87	43.2	11
*Hyparrhinia hirta*	11.45	88.55	18.95	81.05	18.07	64.59	32.1	12
*Rhynchosia minima*	12.21	87.79	19.95	80.05	15.05	36.30	19.8	13
*Cenchrus cilaries*	11.96	88.04	21.64	78.36	20.25	60.85	28.8	14
*Acacia seyal*	14.21	85.79	17.59	82.41	10.6	29.11	27.5	15

Note

% ADF = percent acid detergent fiber, % Ash = percent ash, % CP = percent crude protein, % DM = percent dry matter, % M = percent moisture, % NDF = percent nitrogen detergent fiber, % ODM = percent organic dry matter, FPI = food preference index. Note that all data are on dry matter basis.

### 3.2 Feeding preference

Among the plant species consumed by Bohor reedbuck *Digitaria abyssinica* (1.19 FPI) and *Cynodon dactylon* (1.13 FPI) were the most preferred species during the study period followed by *Panicum maximum* (0.76 FPI), whereas *Acacia seyal* (0.17 FPI) and *Centuries cilaries* (0.19 FPI*)* was the least preferred species (Table 2). Food preference is highly determined by availability and quality of food (Kingdon & Hoffmann, 2013). Mann–Whitney rank test analysis indicated that there is significant correlation between frequency of use of plants consumed and availability (relative frequency of occurrence) (*W* = 180.0, *p* = .03). For instance, *Digitaria abyssinica* is among the least available species, but has the highest crude protein (CP) content (23.75% CP) and contains relatively higher moisture (13.6%) and less fiber (61.19% Neutral Detergent Fiber (NDF) and 27.8% Acid Detergent Fiber (ADF)) (Table 3). Several authors have reported that selective grazing is highly governed by nutritional content such as CP, moisture, NDF and ADF (e.g., Chinomona et al., 2018; Gutbrodt, 2006; Kingdon & Hoffmann, 2013; Omphile et al., 2004). The plants species such as *Cynodon dactylon* (0.89 frequency of usage) and *Chloris gayana* (0.54 frequency of usage) were frequently consumed plant species during both seasons (Table 2). However, *Digitaria abyssinica* (0.86 frequency of usage) and *Dactyoctenium aegyptium* (0.75 frequency of usage) were the most commonly consumed during the wet season. On the other hand, *Pennisetum ciliare* (0.85 frequency of usage) and *Panicum infestum* (0.76 frequency of usage) were frequently consumed during dry season. It has been reported that diet composition of many ungulate species varies substantially among seasons (Ego et al., 2003; Halsdorf, 2011). There was also statistically significant variations in the frequency of each plant species consumed among habitat types during both dry (χ^2^ = 24.23, *df* = 3, *p* = .01) and wet (χ^2^ = 17.27, *df* = 3, *p* = .01) seasons. This is mainly attributed to the change in availability of a particular species and change in nutrition content, a general decline of nutrient content as dry season continues (Ombabi et al., 2001). Particularly, all those species frequently consumed during the dry seasons were available during both seasons. Both *Pennisetum ciliare* and *Panicum infestum* were frequently consumed during the dry seasons mainly due to their highest moisture (11.97 and 11.54% respectively) content (Table 3).

### 3.3 Proximate analysis of plant species

*Balanites aegyptiaca* (17.53%) and *Panicum maximum* (11.66%) had the lowest moisture contents (Table 3). *Digitaria abyssinica* (23.75%) and *Dactyoctenium aegyptium* (21.74%) species had the highest crude protein content, whereas *Cynodon ethiopicus* (10.41%) and *Acacia seyal* (10.59%) had the least nitrogen and crude protein content. The NDF of *Chloris gayana* (69.77%) and *Bothriochloa insculpta* (67.77%) was highest, while was least in *Balanites aegyptiaca* (28.91%) and *Acacia seyal* (29.11%). *Panicum maximum* (49.3%) and *Panicum infestum* (45%) had the highest ADF, while *Rhynchosia minima* (19.8%) and *Acacia seyal* (27.5%) had the least (Table 3).

**FIGURE 2:**
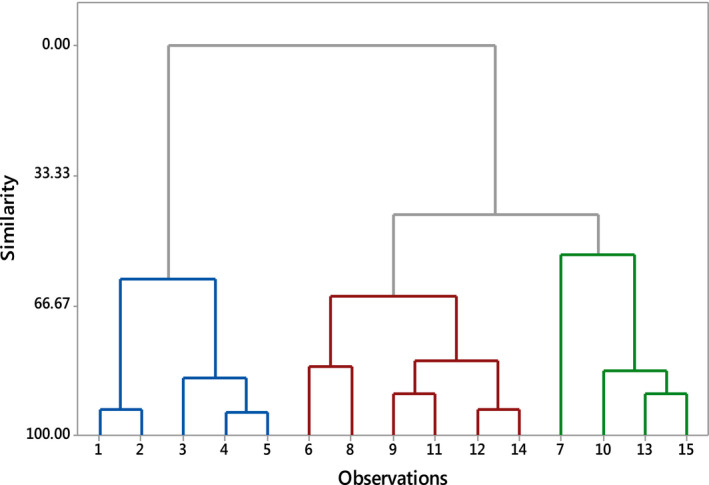
A Dendrogram with Complete Linkage, Euclidean Distance showing cluster observations of plants consumed by Bohor reedbuck based on percent moisture, percent crude protein content, and Percent food preference. Note that Blue cluster of observations at the start of the Dendrogram are plants with higher nutritional quality (percent moisture and crude protein) and with high food preference index, cluster of observation in the middle highlighted red are plants with moderate nutritional quality (percent moisture and crude protein) and food preference and green cluster of observations at the end of the Dendrogram are plants with least nutritional quality (percent moisture and crude protein) and least preferred. Note: 1 = *Digitaria abyssinica*, 2 = *Cynodon dactylon*, 3 = *Panicum maximum*, 4 = *Chloris gayana*, 5 = *Dactyoctenium aegyptium*, 6 = *Panicum infestum*, 7 = *Balanites aegyptiaca*, 8 = *Bothriochloa insculpta*, 9 = *Pennisetum ciliare*, 10 = *Rhus quartiniana*, 11 = *Cynodon aethiopicus*, 12 = *Hyparrhinia hirta*, 13 = *Rhynchosia minima*,14 = *Cenchrus cilaries,* 15 = *Acacia seyal*

The final partition of the cluster group analysis clustered all the observations at 66.7% similarity in to three clusters. Cluster 3 has the highest number of observations (8), while cluster 1 has the least number of observations (2) (Table 4, Figure 2). The three clusters are highlighted in different color in Figure 2.

**TABLE 4:** Summary of statistics of cluster group analysis for the proximate analysis of 15 plant species consumed by Bohor reedbuck, cluster observations using centroids’ distance

Clusters	Number of observations	Within clusters sum of square	Average distance from centroids	Maximum distance from centroids
Cluster 1	2	20.254	3.1823	3.1823
Cluster 2	5	605.737	10.2922	16.0826
Cluster 3	8	593.586	8.0485	12.5933

Note

The clusters are indicated by different colors in the Dendrogram (Figure 2).

The 2 species clustered in cluster one had higher crude protein, moderate NDF and ADF, the 5 species under cluster two had moderate moisture, crude protein highest, NDF and ADF proportion. On the other hand, species under cluster three had the lowest moisture, crude protein, NDF and ADF proportions (Table 5).

**TABLE 5:** Summery table of proximate analysis cluster grouping for 15 plant species at Alage Agricultural College, Ethiopia

Variable	Cluster 1	Cluster 2	Cluster 3	Centroids
%M	12.655	14.55	12.7275	13.3253
%CP	21.155	17.26	15.4525	16.8153
%FP	93.500	61.60	26.3750	47.0667

Note

% *M* = percent moisture, % CP = percent crude protein, % FP = Percent food preference. The centroid represents the “average observation” within a cluster across all the variables (% M, % CP and % FP) in the analysis.

The most preferred species (with relatively highest protein content); *Digitaria abyssinica* and *Cynodon dactylon* followed by *Panicum maximum* and *Chloris gayana* contribute the highest proportion (60.57%) of the diet of Bohor reedbuck. The report from Sudan at Dinder National Park (Bordering Ethiopia) by Ahmed (2005) shows that *Cynodon dactylon* is the first ranked species to be consumed by Bohor reedbuck and also this plant species contributed the highest proportion to the diet of nyala (*Tragelaphus angasi*) in southern Africa (Pienaar, 2013). For herbivores, foraging preferences are shaped by multiple constraints, such as the availability of the food, nutritional quality of the food and the requirements of the animals (Sinclair et al., 2006). The feeding preference of Bohor reedbucks might be influenced by the nutritional quality, especially crude protein and moisture content and frequency of occurrence of the plant species. The first rank preferred plant species by Bohor reedbuck (*Digitaria abyssinica* and *Cynodon dactylon*) illustrate the above statement. *Digitaria abyssinica* was preferred by its high nutritional quality (high crude protein and the lowest ADF) as evidenced by proximate analysis, even though it had the lowest occurrence. However, *Cynodon dactylon* had moderate nutritional quality (18.56% CP and 11.71% moisture) but its high occurrence could make it be the more frequently consumed.

Furthermore, the optimal foraging theory states that animals maximize fitness through a foraging strategy that incur low cost and with maximum energy gain, maximizing the net energy gain. Particularly, *Cynodon dactylon* (0.79 frequency of occurrence) followed by *Panicum maximum* (0.79 frequency of occurrence) and *Chloris gayana* (0.81 frequency of occurrence) are the most available forage species in the area and *Chloris gayana* is the second highest in terms of CP content (Table 4). Hence, a feed with second highest nutritionally quality (*Chloris gayana,* 21.17% CP) and that is readily available (low energy cost) maximize net energy gain and will have high potential for selective grazing. But, frequency of occurrence by itself might not affect the feeding preference of Bohor reedbuck; *Cynodon aethiopicus* demonstrates this. It was the least preferred species despite its highest frequency of occurrence (0.90) in the area. This is possibly due to the lower digestibility and food quality of the plant species; nutritionally this species had the lowest crude protein (10.41%) and the highest ADF (43.2%) that makes it more indigestible and less palatable. Furthermore, the presence of anti‐nutritional factor such as secondary metabolites (flavonoids, tannin etc) determines selective grazing (Reed, 1995). Particularly, *Cynodon aethiopicus* could be least preferred due to its high concentration of toxic substance. It has been reported that C. *aethiopicus* has the potential to produce high levels of prussic acid (HCN) any time during the growing season up to 250 ppm (Harlan et al., 1970). Furthermore, *Rhus quartiniana* had the highest CP content (21.85%), but among the least preferred. This is probably attributed to its high concentration of tannin. It was reported that *R. quartiniana* contains high concentration of tannin in the leaves and barks (Miller et al., 2001).

**FIGURE 3:**
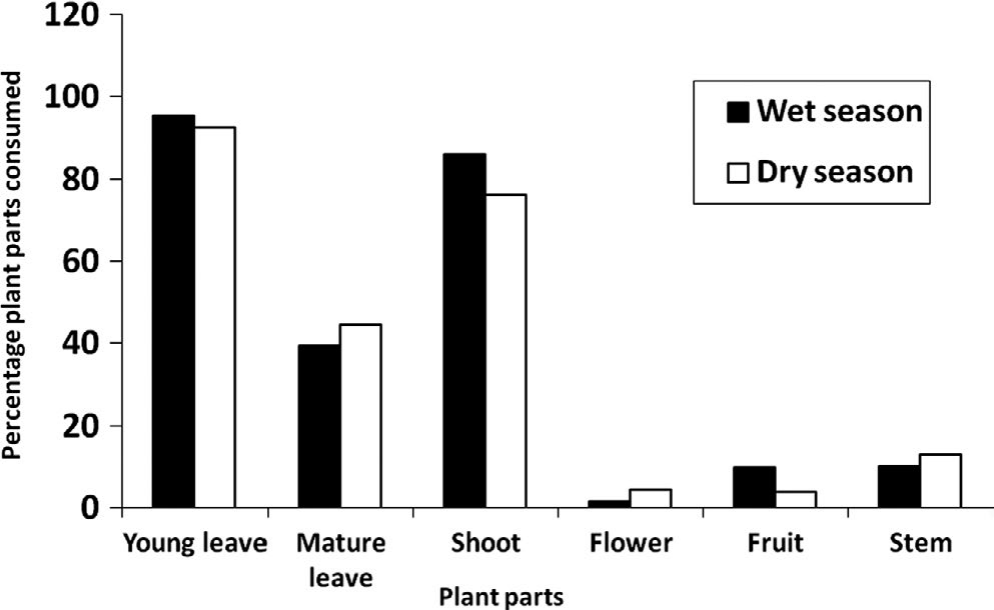
Proportion of plant parts consumed by Bohor reedbuck during wet and dry seasons

Bohor reedbucks preferred to consume young leaves and shoots (comprised over 95% of their consumption) of the plants during the study period, while flowers (1.33%) (few grass species) and fruits (2.90%) (few grass species) of the plant species were the least preferred (Figure 3). Seasonally, Bohor reedbuck's preference to consume young leaves was similar during both wet and dry seasons, but a slight difference was observed with the consumption of other plant parts. There was higher consumption of shoots of plants during the wet season than the dry season whereas leaves consumption increased during the dry season (Figure 3).

Since the majority (74%) of plants selectively grazed by the Bohor reedbuck was grasses, the nutritional analysis constituted mainly leaves shoots and rarely flowers and fruits. For the remaining non‐grass species, only leaves were considered for nutritional analysis. The Bohor reedbuck preference to feed on young leaves and shoots could be due to the fact that green parts of the plant are more nutritious with high moisture content and easily digested due to low‐fiber content as opposed to the dry parts (Woie, 1984). The proximate analysis also indicated that high concentration of nutrients (e.g., CP) in the leaves and shoots for those that were highly preferred. It was reported that the standard range of crude protein ranges from 8% to 30% of dry weight (Huskie et al., 2010). The CP content of the most preferred plant species range above 20% (*Digitaria abyssinica* (23.75% CP, *Chloris gayana* 21.17%). Seasonally, the consumption of stems, flowers and mature leaves of plants by Bohor reedbucks increased during the dry season and also browsing increased during the dry season while availability of feeds decrease. There was a variation in the level of consumption of each plant part among plant habit/growth form and season of the year. For instance, Bohor reedbucks preferred to consume the flowers and shoots of the tree and shrub species especially during the dry season, but they preferred to consume the young leaves and shoots of the herb species in the wet season. Young leaves, shoots, fresh stems, and flowers of grasses (predominant consumed species in the diet composition of the Bohor reedbuck) were more available during wet seasons than dry season. Consequently, more matured leaves, shoots, stems, and flowers were consumed during dry season. Generally, it is known that young leaves and shoot in grass species provide the best quality of food in terms of nutritional content than mature ones and hence preferred. However, if young ones are not available ungulates shift to mature ones. Selective grazing of ungulates is both determined by availability and nutritional quality (Gutbrodt, 2006).

## 4 CONCLUSION

Both plant species availability and nutritional quality determine food preference of Bohor reedbuck. The feeding preference of Bohor reedbucks to graze grass species having high‐quality nutritional profile implies the need for promoting sustainable conservation and growth of those preferred plant species as an input for sustainable conservation of the Bohor reedbucks. Further in‐depth research needs to be done on ecological importance of Bohor reedbuck.

## CONFLICT OF INTERESTS

We the authors declare that we have no competing interests.

## AUTHOR CONTRIBUTION

**Yonas Derebe:** Conceptualization (equal); Data curation (lead); Formal analysis (lead); Funding acquisition (lead); Investigation (equal); Methodology (supporting); Project administration (equal); Resources (lead); Software (supporting); Supervision (supporting); Validation (supporting); Visualization (equal); Writing‐original draft (equal); Writing‐review & editing (supporting). **Zerihun Girma:** Conceptualization (lead); Data curation (supporting); Formal analysis (equal); Funding acquisition (supporting); Investigation (equal); Methodology (lead); Project administration (equal); Resources (supporting); Software (lead); Supervision (lead); Validation (equal); Visualization (equal); Writing‐original draft (equal); Writing‐review & editing (lead).

## Data Availability

I will here confirm that I will avail the data that support the findings of this study up on after publication of the manuscript. Particularly, the data that support the findings will be available in *Bohor reedbuck diet analysis* file name at *Zenodo* data repository following the date of publication.
